# Supplementary material for: Dynamic transcriptomic profiles of zebrafish gills in response to zinc supplementation
Source: BMC Genomics. 2010 Oct 11;11:553. doi: 10.1186/1471-2164-11-553 (PMC3091702; doi:10.1186/1471-2164-11-553)
Supplement: Additional file 2 — Interactive Direct Interaction Network representing the molecular interactions between zinc, copper, iron, calcium and proteins encoded by transcripts changed by zinc supplementation. Mini web-site containing index.html and hyperlinked pages in subdirectory describing a Direct Interaction Network automatically generated based on curated interactions contained within the proprietary PathwayArchitect database. Ovals represent proteins and the circles symbolize metal ions. Objects are coloured by their abundance in zebrafish at the time-point they were significantly different from the control is a scale from -4 fold (dark green) to +4 fold (dark red). Where significant differences were found at more than one time-point, the colour overlay shows expression at the first instance. Dark blue squares denote 'binding', and light blue squares 'expression'; green squares stand for 'regulation', green diamonds for 'metabolism', and green circles for 'promoter binding'. Arrow heads indicate directionality of the interaction where annotated. All nodes and edges can be further interrogated by selecting the relative area of the image. [file 1471-2164-11-553-S2.zip › PathwayArchitect Zn xs DIN/145545.html]

# PROTEIN: CEBPG

|  |  |
| --- | --- |
| Name | CEBPG |
| Type | PROTEIN |
| Description | CCAAT/enhancer binding protein (C/EBP), gamma |
| Note | The C/EBP family of transcription factors regulates viral and cellular CCAAT/enhancer element-mediated transcription. C/EBP proteins contain the bZIP region, which is characterized by two motifs in the C-terminal half of the protein: a basic region involved in DNA binding and a leucine zipper motif involved in dimerization. The C/EBP family consist of several related proteins, C/EBP alpha, C/EBP beta, C/EBP gamma, and C/EBP delta, that form homodimers and that form heterodimers with each other. CCAAT/enhancer binding protein gamma may cooperate with Fos to bind PRE-I enhancer elements. |
| Alias | CCAAT/enhancer binding protein ,gamma |
|  | C/EBP[g] |
|  | Gpe1bp |
|  | CEBPG |
|  | C/EBP |
|  | G-CSF promoter element 1 binding protein |
|  | Cebpg |
|  | Rat homolog to a human CCAAT/enhancer binding protein - gamma |
|  | GPE1-BP |
|  | Ig/EBP |
|  | CCAAT/enhancer binding protein gamma |
|  | GPE1-BP (C/EBP gamma) |
|  | GPE1BP |
|  | CEBPRNA |
|  | GPE1-binding protein |
|  | C/EBP gamma |
|  | Granulocyte colony-stimulating factor promoter element 1 binding protein |
|  | C/EBPgamma |
|  | IG/EBP-1 |
|  | Immunoglobulin enhancer binding protein 1 |


---

|  |  |
| --- | --- |
| GO Component | nucleus |


---

|  |  |
| --- | --- |
| GO ID | GO:0005634 |
|  | GO:0003677 |
|  | GO:0006355 |
|  | GO:0046982 |
|  | GO:0003690 |
|  | GO:0016563 |
|  | GO:0042803 |
|  | GO:0006357 |
|  | GO:0006350 |
|  | GO:0016564 |
|  | GO:0003700 |


---

|  |  |
| --- | --- |
| MIM | MIM:138972 |


---

|  |  |
| --- | --- |
| Connectivity | 269 |


---

|  |  |
| --- | --- |
| Entrez ID | 25301 |
|  | 12611 |
|  | 1054 |


---

|  |  |
| --- | --- |
| Agilent ID | A\_53\_P165385 |
|  | A\_51\_P521216 |
|  | A\_23\_P208801 |
|  | A\_14\_P129724 |
|  | A\_44\_P1030594 |
|  | A\_23\_P356755 |
|  | A\_52\_P121675 |
|  | A\_42\_P645196 |
|  | A\_44\_P299451 |
|  | A\_52\_P438082 |


---

|  |  |
| --- | --- |
| Cellular Localization | Nucleus |
|  | Organelle |
|  | Cell |


---

|  |  |
| --- | --- |
| Pathway | Zn xs inventory |
|  | Zn xs DIN |


---

|  |  |
| --- | --- |
| GO Process | regulation of transcription, DNA-dependent |
|  | regulation of transcription from RNA polymerase II promoter |
|  | transcription |


---

|  |  |
| --- | --- |
| UniGene | Rn.10332 |
|  | Mm.273090 |
|  | Hs.429666 |


---

|  |  |
| --- | --- |
| Affymetrix Probeset ID | 113580\_at |
|  | 1371038\_at |
|  | 1372885\_at |
|  | 1380961\_at |
|  | 1425261\_at |
|  | 1425262\_at |
|  | 1451639\_at |
|  | 165100\_i\_at |
|  | 204203\_at |
|  | 225527\_at |
|  | 39219\_at |
|  | 54985\_at |
|  | 58738\_at |
|  | 92195\_at |
|  | g4502768\_3p\_at |
|  | 135139\_at |
|  | Hs.7887.0.A1\_3p\_at |
|  | rc\_AA818253\_at |
|  | U20240\_at |
|  | X55499\_s\_at |
|  | X64403\_at |
|  | RC\_AA179189\_s\_at |
|  | RC\_AA256952\_at |
|  | TC35150\_s\_at |
|  | TC40298\_at |


---

|  |  |
| --- | --- |
| GO Function | double-stranded DNA binding |
|  | transcriptional repressor activity |
|  | transcriptional activator activity |
|  | transcription factor activity |
|  | protein heterodimerization activity |
|  | DNA binding |
|  | protein homodimerization activity |


---

|  |  |
| --- | --- |
| Nucleotide | NM\_009884 |
|  | BC013128 |
|  | AB012275 |
|  | AI847655 |
|  | BC011319 |
|  | X55499 |
|  | CB993585 |
|  | BC007582 |
|  | BT019819 |
|  | AK169583 |
|  | X64403 |
|  | U20240 |
|  | AK171859 |
|  | NM\_001806 |
|  | AK159544 |
|  | S80556 |
|  | AB012273 |
|  | BT019820 |
|  | AK194059 |
|  | AK050634 |
|  | XM\_341842 |
|  | AK157391 |


---

|  |  |
| --- | --- |
| Protein | BAE41241 |
|  | BAA25312 |
|  | P53568 |
|  | CAA45745 |
|  | BAE34076 |
|  | AAH13128 |
|  | BAE35170 |
|  | AAH11319 |
|  | CAA39116 |
|  | AAC50201 |
|  | NP\_001797 |
|  | XP\_341843 |
|  | P26801 |
|  | BAA25311 |
|  | P53567 |
|  | AAH07582 |
|  | NP\_034014 |
|  | AAV38623 |
|  | AAV38622 |
|  | BAE42702 |
|  | BAC34355 |


---

|  |  |
| --- | --- |
| Organism | Mammal |


---

|  |  |
| --- | --- |
| Location | chromosome 1, 1q21 (Rattus norvegicus) |
|  | chromosome 7, 7 B1 (Mus musculus) |
|  | chromosome 19, 19q13.11 (Homo sapiens) |


---

|  |  |
| --- | --- |
